# Adverse drug events in hospitalized COVID-19 patients: a pharmacovigilance perspective on ICU management and fentanyl exposure

**DOI:** 10.3389/fepid.2026.1770145

**Published:** 2026-08-18

**Authors:** Michele Ferreira Murgel, Marcel de Souza Borges Quintana, Roberta Olmo Pinheiro, Vítor Santos da Silva, Cláudia Luciana dos Santos Moura, Vanessa da Gama Oliveira, Cláudia Maria Valete Rosalino, Gilberto Marcelo Sperandio da Silva

**Affiliations:** 1 Evandro Chagas National Institute of Infectious Diseases (INI), FIOCRUZ, Rio de Janeiro, Brazil; 2 Leprosy Laboratory, Oswaldo Cruz Institute, Oswaldo Cruz Foundation, Rio de Janeiro, Brazil; 3 Department of Otorhinolaryngology and Ophthalmology, Federal University of Rio de Janeiro (UFRJ), Rio de Janeiro, Brazil

**Keywords:** COVID-19, fentanyl, mechanical ventilation, naranjo algorithm, opioid sedation, pharmacovigilance, polypharmacy

## Abstract

**Background:**

Pharmacovigilance is the science and practice aimed at detecting, assessing, understanding, and preventing adverse drug events (ADEs). During the COVID-19 pandemic, a wide range of medications - suchasantivirals, corticosteroids, anticoagulants, immunomodulators, and sedatives - were used during respiratorysupport. Reports of hepatotoxicity, cardiotoxicity, renal impairment, and neurological effects underscored the need for systematic safety monitoring. Since 2012, our research group has been monitoring the clinical use of various prescriptions at the National Institute of Infectious Diseases in Brazil. In this study, we aimed to evaluate factors associated with adverse drug events in hospitalized COVID-19 patients,with emphasis on ICU management practices, polypharmacy and fentanyl exposure.

**Methods:**

This observational study was conducted between March and September 2021 and included patients with a positive molecular test for SARS-CoV-2. Sociodemographic characteristics, including age, sex, educational level, and family income, as well as medical history such as hypertension, diabetes, obesity, dyslipidemia, fever, cough, dyspnea, and anosmia, were collected. Clinical data on hospitalizations, commonly prescribed medications, length of stay in intensive care units, vaccination status, oxygen supplementation, type of respiratory support, deaths, and oxygen saturation were also recorded. All adverse reactions were classified using a validated algorithm and standard terminology, and patients were followed until discharge or death. Data were initially collected on paper forms and subsequently entered into an electronic data capture platform. Associations between patient characteristics, treatments, and adverse reactions were analyzed using Poisson regression, with statistical significance set at *p*-value < 0.05.

**Results:**

Among 113 patients, 110 experienced at least one adverse event, totaling 411 events. The mean age was 56.5 years; 54.9% were male, 53.1% had hypertension, 28.3% had diabetes, 22.2% had received at least one vaccine dose, and 17.7% died. Higher rates of adverse events were observed among intubated patients and those who received fentanyl, which is commonly used during the intubation process.

**Conclusions:**

In this study, intubation and fentanyl use were associated with higher rates of ADEs. These findings should be interpreted cautiously given the severity of illness, ICU sedation practices, and the possibility of confounding by indication. Therefore, active pharmacovigilance strategies may contribute to improved medication safety in critically ill populations.

## 1 Introduction

The outbreak of COVID-19 resulted from the emergence of the Severe Acute Respiratory Syndrome Coronavirus 2 (SARS-CoV-2). The virus was first identified in Wuhan, China, before expanding globally to cause a pandemic. In this context, the mortality rate was 2.3% and was higher among those suffering from preexisting comorbid conditions such as cardiovascular disease, diabetes, chronic respiratory disease, hypertension, and cancer (1, 2). Additionally, several drugs have been used in hospitalized patients to treat and control infectious and inflammatory complications resulting from COVID-19, including antibiotics, corticosteroids, anticoagulants, and antiretrovirals (2–6).

Data from the literature indicate that patients with COVID-19 receiving pharmacological treatment are at risk of developing Adverse Drug Events (ADEs) (7, 8), with some drugs, such as fentanyl, associated with serious outcomes. Fentanyl was widely used to manage severe respiratory symptoms in COVID-19 due to its effectiveness in reducing dyspnea and pain; however, its use requires close monitoring given the risk of respiratory depression, particularly in patients with preexisting comorbidities. Reported adverse effects, including acute lung injury and alveolar hemorrhage, further emphasize the need for careful opioid management in this setting (9). Other studies suggest that drugs acting on the central nervous system in COVID-19 patients may also contribute to ADEs (10–12).

Previous work from our group at the Evandro Chagas National Institute of Infectious Diseases (INI, Fiocruz, Brazil)—a national reference center for the management of severe COVID-19—demonstrated the feasibility of using sociodemographic and clinical characteristics in multivariate models to investigate ADEs (13). In the context of the COVID-19 pandemic, critically ill patients were frequently exposed to invasive ventilation, prolonged hospitalization, sedation protocols and extensive polypharmacy. Accordingly, this prospective observational study primarily aimed to identify sociodemographic and clinical factors associated with ADEs in hospitalized patients. Additionally, we explored the role of medications commonly used in intensive care settings, particularly fentanyl, given its frequent use during invasive mechanical ventilation and Intensive Care Unit sedation protocols.

## 2 Materials and methods

### 2.1 Ethical approval

This observational, prospective, longitudinal study was conducted in accordance with the Declaration of Helsinki and was approved by the Research Ethics Committee of the Evandro Chagas National Institute of Infectious Diseases (INI/FIOCRUZ), under approval number 4.560.734 (CAAE: 42119321.7.0000.5262). All patients or their legal representatives provided informed consent for their participation in the study

### 2.2 Study design and setting

The present study was an observational, prospective, longitudinal investigation involving individuals with COVID-19 who were hospitalized between March and September 2021 at the Evandro Chagas National Institute of Infectious Diseases (INI-Fiocruz), Oswaldo Cruz Foundation, Rio de Janeiro, Brazil. All patients included in this study tested positive for COVID-19, and the diagnosis was confirmed by SARS-CoV-2 detection using Real-Time Reverse Transcription PCR (RT-PCR) (Biomanguinhos kit E + P1], Fiocruz, Brazil) performed on nasopharyngeal secretions and/or bronchoalveolar fluid. This study included all eligible patients admitted during the study period, characterizing a census of the target population rather than a sample-based design. Therefore, a formal sample size calculation was not performed.

### 2.3 Patient recruitment and follow-up

Contact with patients’ families for screening and authorization to participate was conducted via telemedicine. All patients were observed from hospital admission until discharge or death.

### 2.4 Variables collected

The variables collected included sex, age, number of medications, length of hospitalization (scaled in 10-day units), type of hospitalization, educational level, comorbidities (such as systemic arterial hypertension, diabetes mellitus, dyslipidemia, and obesity), family income, vaccination status, respiratory support, patient outcomes (discharge, transfer, or death), and occurrence of ADEs. Clinical data such as symptoms (fever, cough, dyspnea, nausea/vomiting, anosmia), laboratory tests, medical diagnoses, and ICD-10 classifications were also recorded.

### 2.5 Data collection and classification of ADEs

The REDCap platform was used to collect and manage study data. The causality of ADEs was assessed using Naranjo's algorithm, which classifies events as possible, probable, or doubtful (14). ADEs were further classified according to the World Health Organization Adverse Reaction Terminology (WHO-ART) System Organ Class (15).

### 2.6 Data analysis

The exploratory analysis of the data consisted of calculating means, standard deviations, and frequencies of the variables of interest. As certain determinants influenced the total number of adverse events, a Poisson regression model was applied, as no overdispersion was detected. The effects of the models were analyzed by exponentiating the coefficients, which were interpreted as the Rate Ratios (RR) of adverse events observed during the study period.

To build the multivariable model, we defined two groups of candidate predictors to assess their joint impact on adverse events. The first group included demographic and clinical variables (e.g., sex, education level), and the second group included the use of specific medications (e.g., enoxaparin, dipyrone). This grouping was defined *a priori*, as some medications may be specific to certain clinical conditions or comorbidities and could introduce collinearity. Within each group, univariate analyses were performed using Poisson regression, as preliminary assessments indicated no evidence of overdispersion. Variables with p-values < 0.20 (Wald test) were selected as candidates for the multivariable model. Before inclusion, correlation among predictors was evaluated based on clinical knowledge and inspection of correlations between variables; in cases of potential correlation, only one variable was retained based on clinical relevance. A multivariable model was then fitted including all candidate variables, and a backward elimination procedure based on Wald test p-values was applied iteratively, retaining variables with p-values < 0.05 in the final model. Model assumptions were further assessed by evaluating overdispersion through fitting a Negative Binomial model, and model fit was compared using the Akaike Information Criterion (AIC). The Poisson model demonstrated better performance and was therefore retained as the final model. All analyses were performed using R software version 4.2.2. Additionally, clinically relevant variables such as age and disease severity indicators were considered in the modeling strategy to reduce potential confounding effects.

## 3 Results

**Figure 1:**
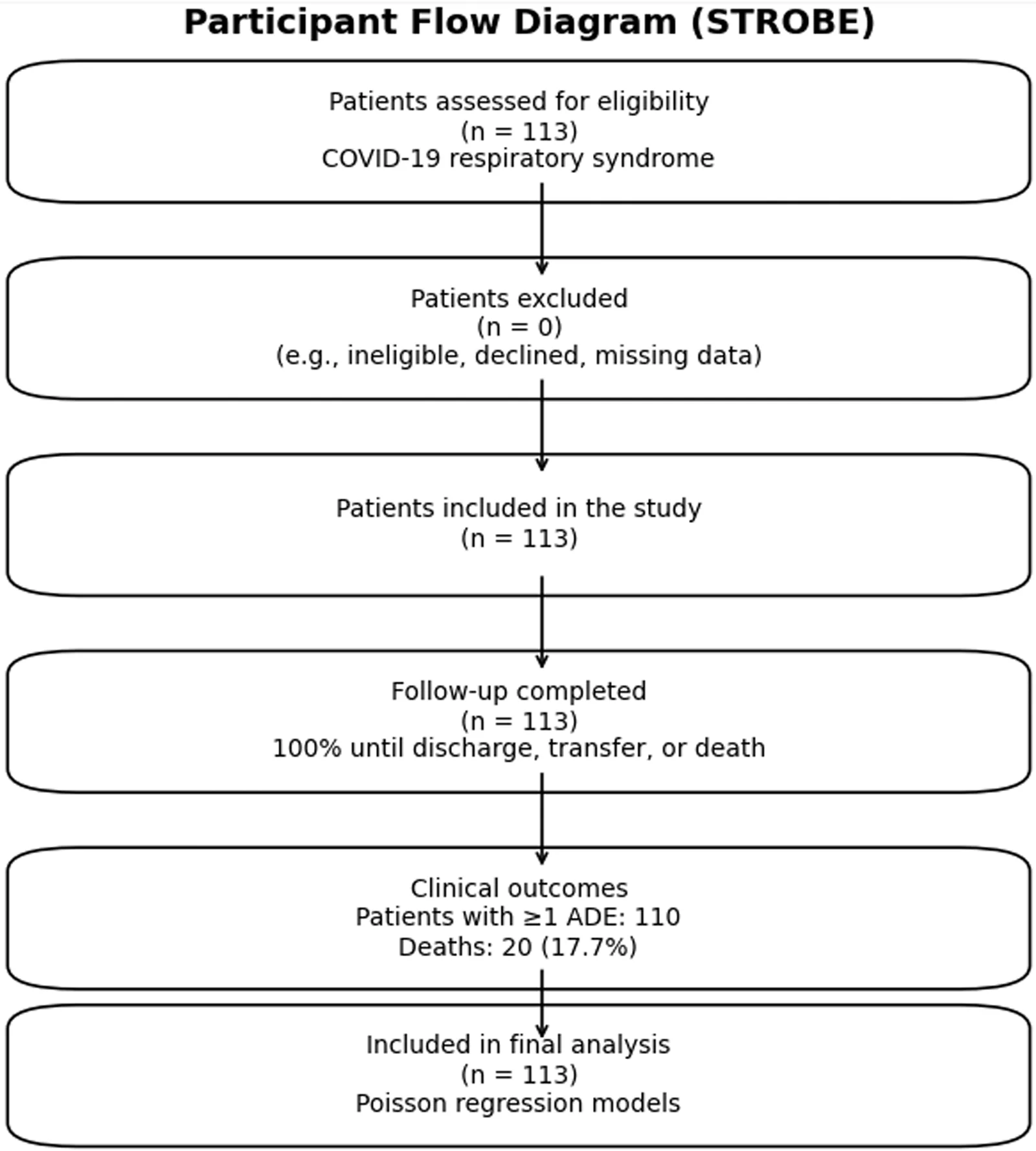
Participant flow diagram (STROBE). This diagram summarizes the progression of participants through the study, from eligibility assessment to final analysis. All 113 patients were included, completed follow-up, and contributed outcome data, including 110 with at least one ADE and 20 deaths (17.7%).

A total of 113 patients with confirmed COVID-19 were included in the study. Most participants were male (54.9%), with a mean age of 56.5 years. The cohort predominantly had more than nine years of formal education, and 27% reported a household income equivalent to two minimum wages. Among all participants, 110 experienced at least one adverse drug event (ADE), and 20 patients (17.7%) died during hospitalization. The overall flow of participants throughout the study is summarized in Figure 1. The average length of hospital stay was 14.6 days, and the average number of medications used was 10.9. There was a high prevalence of comorbidities, including systemic arterial hypertension (53.1%), diabetes mellitus (28.3%), obesity (37.2%), and dyslipidemia (13.3%). In this cohort of 113 patients, 30.9% were vaccinated, 22.1% were intubated, and 17.7% died. In this study, 411 ADEs were identified, of which 98.3% were classified as possible and 1.7% as probable (Table 1).

**Table 1:** Demographics and clinical characteristics of COVID-19 patients admitted to the hospital center COVID-19 – FIOCRUZ (*N* = 113).

Demographic/Clinical Variables	Frequency/Percentage n (%)
Sex: Male	62 (54.9)
Age^a^	56.5 (17.2)
Number of medications^a^	10.9 (5.7)
Hospitalization days^a^	14.6 (23.9)
Level of Education: ≥ 9 years	46 (51.7)
Systemic Arterial Hypertension	60 (53.1)
Diabetes Mellitus	32 (28.3)
Dyslipidemia	15 (13.3)
Obesity	42 (37.2)
Family Income: One Minimun wage	24 (20.9)
Family Income: Two Minimun wage	31 (27)
Family Income: Three Minimun wage	11 (9.6)
Family Income: > Three Minimun wage	20 (17.4)
Family Income: Unreported	27 (25.1)
Vaccine: Any	35 (30.9)
Vaccine: Unreported	41 (27.3)
Respiratory Support: Oxygen catheter	51 (45.1)
Respiratory Support: Oxygen mask	18 (15.9)
Respiratory Support: Intubation	25 (22.1)
Death	20 (17.7)

a Quantitative variables are presented as mean (SD).

To identify the factors associated with the occurrence of adverse events, a Poisson regression model was applied. Table 2 presents the absolute and relative frequencies of each variable, as well as the crude and adjusted rate ratios of ADEs for each predictor, along with their corresponding 95% confidence intervals (CIs) and p-values. Overdispersion was assessed using the ratio of residual deviance to residual degrees of freedom from the Poisson regression model. Model comparison showed similar fit between models (Negative binomial AIC = 447.64; Poisson AIC = 445.64), therefore the Poisson model was retained. The estimated ratio was 0.88, indicating no evidence of substantial overdispersion and supporting the adequacy of the Poisson distribution assumption. Consistently, the Negative Binomial model did not improve model fit compared with the Poisson model (Negative binomial AIC: 447.64; Poisson AIC 445.64; Poisson retained). No offset was used for the ADE count outcome. In the univariate analysis, variables such as respiratory support [RR for intubation (95% CI) = 1.61 (1.19–2.21); *p* < 0.01), vaccine doses [RR for two doses (95% CI) = 1.36 (1.01–1.83); *p* = 0.04]; age [RR (95% CI) = 1.01 (1.00–1.01); *p* = 0.03], O₂ saturation [RR (95% CI) = 0.99 (0.98–1.00); *p* = 0.03], and each 10 hospitalization days [RR (95% CI) = 1.04 (1.01– 1.08); *p* = 0.01] demonstrated significant associations with adverse events. However, in the multivariate analysis, only respiratory support [RR for intubation (95% CI) = 1.65 (1.21–2.26); *p* < 0.01] and each 10 days of hospitalization [RR (95% CI) = 1.04 (1.00–1.08); *p* = 0.0428] remained statistically significant. In this context, the examination of respiratory support data revealed that the rate of total adverse events was 65% higher among individuals who underwent intubation compared with those who did not require respiratory support, and that for every 10 days of hospitalization, the rate of total adverse events increased by 3.8% (Table 2).

**Table 2:** Association of risk factors with ADEs using univariate and multivariate poisson regression in patients hospitalized with COVID-19 (*N* = 113).

Variables	n (%)	Crude RR (95% CI)	*p*-value	Adjusted RR (95% CI)	*p*-value
Sex: Female	51 (45.1)	1.12 (0.92–1.36)	0.25		
Level of Education: >=9	46 (51.7)	0.93 (0.75–1.16)	0.51		
Respiratory support: Intubation	25 (22.1)	1.61 (1.19–2.21)	<0.01	1.65 (1.21–2.26)	<0.01
Respiratory support: Oxygen mask	18 (15.9)	1.15 (0.8–1.63)	0.45	1.20 (0.84–1.72)	0.31
Respiratory support: Oxygen catheter	51 (45.1)	1.03 (0.77–1.40)	0.85	1.12 (0.82–1.54)	0.47
Fever	78 (69)	0.92 (0.75–1.13)	0.41		
Cough	80 (70.8)	0.94 (0.77–1.17)	0.59		
Dyspnea	81 (71.7)	1.15 (0.93–1.44)	0.21		
Nausea/Vomiting	22 (19.5)	0.94 (0.73–1.20)	0.62		
Anosmia	11 (9.7)	0.73 (0.49–1.04)	0.10		
Form of hospitalization: ICU	110 (97.3)	1.22 (0.67–2.55)	0.56		
Systemic Arterial Hypertension	60 (53.1)	1.11 (0.92–1.35)	0.29		
Diabetes Mellitus	32 (28.3)	0.94 (0.75–1.16)	0.56		
Dyslipidemia	15 (13.3)	1.03 (0.77–1.35)	0.83		
Obesity	42 (37.2)	0.96 (0.78–1.17)	0.70		
Any vaccine	35 (48.6)	1.25 (0.98–1.59)	0.07		
Vaccine doses: 1	16 (22.2)	1.11 (0.81–1.51)	0.51		
Vaccine doses: 2	15 (20.8)	1.36 (1.01–1.83)	0.04		
Age^a^	56.5 (17.2)	1.01 (1.00–1.01)	0.03		
O2 saturation	93.3 (7.6)	0.99 (0.98–1.00)	0.03		
Hospitalization days^a^	14.6 (23.9)	1.04^b^ (1.01–1.08)	0.01	1.04^b^ (1.00–1.08)	0.04

a Quantitative variables are presented as mean (SD).

b Per 10 hospitalization days.

Poisson regression was also applied to evaluate drugs associated with ADEs in the exposed population. Among the 142 drugs evaluated, omeprazole capsules, fentanyl, midazolam, and noradrenaline significantly increased the incidence of ADEs. Subsequently, in the multivariate analysis, only fentanyl remained statistically significant [RR (95% CI) = 1.62 (1.31–1.99); *p* < 0.01] (Table 3). Overdispersion was assessed using the ratio of residual deviance to residual degrees of freedom from the Poisson regression model. The estimated ratio was 0.87, indicating no evidence of substantial overdispersion and supporting the adequacy of the Poisson distribution assumption. Consistently, the Negative Binomial model did not improve model fit compared with the Poisson model (Negative binomial AIC: 442.28; Poisson AIC 440.28; Poisson retained). The observed associations should be interpreted with caution, as this study design does not allow causal inference. In particular, the relationship between fentanyl use and adverse drug events may reflect confounding by indication, since patients receiving fentanyl were more likely to be critically ill. This is reinforced by the fact that, after combining the two multivariable models into a single model containing respiratory support, days of hospitalization, and fentanyl use, none of the three predictors remained significant, likely because high correlation and overlapping clinical indications (intubation, sedation, and longer hospital stay) attenuated their effects.

**Table 3:** Association of drugs with ADEs evaluated by univariate and multivariate poisson regressions.

Drug/Medication	n (%)	Crude RR (95% CI)	*p*-value	Adjusted RR (95% CI)	*p*-value
Enoxaparin	81 (71.7)	1.14 (0.91–1.42)	0.26		
Dipyrone	74 (65.5)	1.01 (0.82–1.24)	0.93		
Ceftriaxone	56 (49.6)	1.12 (0.92–1.36)	0.25	-	-
IV Dexamethasone	55 (48.7)	1.02 (0.84–1.24)	0.85		
IV Omeprazole ^a^	53 (46.9)	0.89 (0.73–1.08)	0.24		
Omeprazole capsule^a^	47 (41.6)	1.27 (1.04–1.54)	0.02		
Bromopride	42 (37.2)	1.11 (0.91–1.35)	0.30		
Ringer Solution	40 (35.4)	1.26 (1.04–1.53)	0.02		
Heparin	31 (27.4)	1.07 (0.86–1.32)	0.56		
Ipratropium	30 (26.5)	0.99 (0.79–1.22)	0.90		
Prednisone	28 (24.8)	0.91 (0.72–1.14)	0.43		
Dexamethasone capsule	28 (24.8)	1.22 (0.98–1.51)	0.07	-	-
Fentanyl	24 (21.2)	1.62 (1.31–1.99)	<0.01	1.62 (1.31–1.99)	<0.01
Midazolam	22 (19.5)	1.62 (1.31–1.99)	<0.01		
Glucose	21 (18.6)	1.34 (1.06–1.67)	0.01		
Losartan	20 (17.7)	0.91 (0.69–1.17)	0.46		
Noradrenaline	19 (16.8)	1.57 (1.25–1.96)	<0.01		
Dexmedetomidine	18 (15.9)	1.26 (0.98–1.60)	0.07		
Metformin	18 (15.9)	0.96 (0.72–1.24)	0.74		
Insulin	17 (15)	1.24 (0.96–1.58)	0.09		

a Formulations modeled separately due to potential differences in risk profiles.

The data suggest signs, symptoms, and pharmacological changes that may reflect ADEs, including hepatic, renal, gastrointestinal, metabolic, and general systemic disorders. These reactions were classified according to WHO-ART. The most frequent reactions were metabolic and nutritional disorders (38.2%; code 800 – WHO-ART) (Table 4).

**Table 4:** Frequency of signs/symptoms or pharmacological changes (*N* = 411) that may represent ADEs per patient (*N* = 113) at the COVID-19 hospital center according to system organ class and medications most frequently indicating ADEs.

Code	Class	ADEs n(%)	ADEs Cumulative (%)	Medications Most Frequently Indicating ADEs n(%)	Total medications most frequently indicating ADEs *n* (%)
800		159 (38.7)	38.7	Omeprazole 16 (17.7)	90 (100)
				Dipyrone 5 (5.5)	
	Metabolic and			Fentanyl 3 (3.3)	
	nutritional disorders			Dexamethasone 8 (8.9)	
				Prednisone 4 (4.4)	
				Insulin 3 (3.3)	
				Metformin 3 (3.3)	
700	Liver and biliary system disorders	80 (19.5)	58.2	Fentanyl 4 (14.8)	27 (100)
				Prednisone 3 (11.1)	
				Losartan3 (11.1)	
				Midazolam 2 (7.4)	
				Dipyrone 2 (7.4)	
1,810	Body as a whole general disorders	64 (15.6)	73.8	Prednisone 4 (8.3)	48 (100)
				Dexamethasone 3 (6.2)	
				Bromopride 3 (6.2)	
				Fentanyl 2 (4.2)	
				Dexmedetomidine 2 (4.2) Midazolam 5 (10.4)	
				Omeprazole 5 (10.4)	
				Ceftriaxone 3 (6.2)	
				Bromopride 2 (4.2)	
				Losartan 2 (4.2)	
				Metformin 2 (4.2)	
1,300	Urinary system disorders	50 (12.2)	86	Dexamethasone 4 (12.1)	33 (100)
				Omeprazole 3 (9.1)	
				Losartan 3 (9.1)	
				Bromopride 2 (6.1)	
600	Gastro-intestinal system disorders	17 (4.1)	90.1	Fentanyl 4 (17.4)	23 (100)
				Dexmedetomidine 3 (13.0)	
	Others	41 (8.9)	100		

## 4 Discussion

Based on a cohort of 113 patients admitted to the COVID-19 Hospital Center, the socioeconomic and clinical characteristics, as well as the main medications used by this population, were evaluated. The mean duration of hospitalization was 14.6 days (SD = 23.9) and in total, 20 patients (17.7%) died The mean age (60 ± 19 years) and proportion of male patients (59.6%) reported by Matsuzawa *et al*. were similar to observed in our cohort (16).

The main comorbidities identified in our study were hypertension, obesity, and diabetes mellitus. These data are corroborated by a systematic review of 27 articles including 22,753 patients, which identified hypertension (27.4%) and diabetes (17.4%) as the main comorbidities. Another study suggested that obesity is associated with increased severity of COVID-19. and the risk of death (17, 18).

In Brazil, vaccination began with the elderly population, which may have contributed to greater protection for this group during the pandemic (19). The patients in this study were polymedicated (mean 10.9 drugs; SD = 5.7), a finding explained by the high frequency of comorbidities in our cohort. Similar results were observed by Galiza *et al*., who highlighted the need to control excessive medication use in patients with comorbidities, especially among the elderly. Vigilant prescription management, particularly by avoiding the simultaneous use of five or more drugs, is a key strategy to reduce the risk of ADEs. Thus, minimizing polypharmacy is critical to lowering ADE risk (20, 21). Under these circumstances, the coexistence of severe COVID-19, prolonged ICU stay, invasive ventilation, and extensive polypharmacy creates a highly complex clinical scenario for ADE occurrence. In this scenario, patients are simultaneously exposed to sedatives, vasoactive drugs, corticosteroids, anticoagulants, antibiotics, and metabolic support therapies, increasing the possibility of drug interactions and cumulative toxicities. This context reinforces the importance of continuous pharmacovigilance strategies in critically ill hospitalized populations.

The assessment of causality of ADEs showed that 98.3% were classified as possible and 1.7% as probable, according to the Naranjo algorithm. These results indicate that the most frequent causality classification for ADEs in this cohort was “possible,” a finding corroborated by Melo *et al*., who also reported that the highest frequency was possible (67.4%) (10).

Data from the present study indicate that symptoms such as fever, cough, anosmia, and nausea were frequent in patients with COVID-19, corroborating findings by Zhu *et al*. (2). In the univariate analysis of clinical characteristics, the variables age [*p* = 0.03, RR (95% CI) = 1.01 (1–1.01)], oxygen saturation [*p* = 0.03, RR (95% CI) = 0.99 (0.98–1)], days of hospitalization [*p* = 0.01, RR (95% CI) = 1 (1–1.01)] and intubation [*p* = 0.01, RR (95% CI) = 1.61 (1.19–2.21)] were statistically significant for the occurrence of ADEs. However, in the multivariate analysis, only respiratory support [RR intubation (95% CI) = 1.65 (1.21–2.26); *p* < 0.01] and days of hospitalization [RR (95% CI) = 1 (1–1.01); *p* = 0.04] remained statistically significant. In this context, the analysis of respiratory support data revealed that the rate of total adverse events was 65% higher among individuals who underwent intubation compared to those who did not require respiratory support, and for 10 days of hospitalization, the rate of total adverse events increased by 3.8%. According to researchers, patients with invasive ventilation were at greater risk of severe outcomes compared to those who did not use this type of respiratory support, corroborating our findings regarding intubated patients (22, 23).

The univariate association with two vaccine doses did not persist after adjustment, likely reflecting residual confounding and incomplete vaccine data. In the univariate analysis of drug use and adverse events, patients who used fentanyl had 65%, midazolam 59%, and noradrenaline 57% more adverse events than those who did not. However, in the multivariate regression analysis, only fentanyl was statistically significantly associated with ADEs. However, these findings should be interpreted within the context of severe COVID-19 management, ICU sedation practices and potential confounding by indication. Importantly, since fentanyl is frequently administered during invasive mechanical ventilation, the observed association may partially reflect disease severity, prolonged ICU stay and exposure to multiple medications rather than direct causal effects of fentanyl of fentanyl alone (24, 25).

The data from this study also indicate a higher frequency of metabolic disorders (38.7%) and hepatic disorders (19.5%) as signs or symptoms that may represent ADEs in hospitalized patients. According to researchers, some medications can cause hepatic and metabolic injury, corroborating our findings (26, 27). The high prevalence of ADEs observed in this cohort should be interpreted considering the operational definition adopted in this study. In this setting, the adverse events included not only clinically severe reactions but also laboratory abnormalities, metabolic disturbances, gastrointestinal manifestations, and other signs or symptoms identified during active pharmacovigilance surveillance. In critically ill patients with COVID-19, who were frequently exposed to polypharmacy and prolonged hospitalization, the likelihood of detecting multiple concurrent events substantially increased. Furthermore, the prospective monitoring strategy employed in this study may have contributed to higher detection rates compared with studies based exclusively on spontaneous reporting systems. Therefore, these findings likely reflect both the complexity of disease management and the sensitivity of active surveillance approaches.

Finally, considering that this project was developed during the pandemic, when the movement of people was limited by quarantine, telemedicine represented an important resource for screening patients with COVID-19 and facilitating the participation of families in this study. The high prevalence of metabolic and hepatic ADEs, compounded by polypharmacy in comorbid patients, illustrates the clinical management challenges in severe COVID-19. These findings necessitate robust pharmacovigilance systems for real-time drug safety monitoring. Future research priorities include developing predictive ADE risk models for critically ill populations and validating safer sedation protocols to guide clinical practice during pandemic responses. Regarding the study's limitations, it is possible to state that many patients were intubated, and it is likely that some data regarding vaccinations were underestimated, as part of this information was obtained based on reports from family members. Additionally, because multiple comparisons were performed across 142 medications, the observed drug associations should be interpreted as exploratory and hypothesis generating rather than confirmatory findings. Importantly, the extreme workload on healthcare professionals could have led to the under-reporting of less severe adverse events. Similarly, incomplete medical records and the lack of comprehensive medication histories for patients transferred from other facilities may have resulted in an underestimation of some risk factors.

## 5 Conclusions

Patients in this cohort most frequently presented comorbidities such as hypertension, diabetes, and obesity. These conditions are associated with the use of antihypertensive and hypoglycemic drugs, which were among the most prescribed to hospitalized patients during this period. Patients diagnosed with COVID-19 who had comorbidities often used more than five medications, characterizing polypharmacy. Minimizing polypharmacy is an important patient safety strategy.

In this study, intubation and fentanyl use were associated with higher rates of adverse drug events. However, these findings should be interpreted within the context of severe COVID-19 management, ICU sedation practices, and potential confounding by indication. Since fentanyl is frequently used during invasive mechanical ventilation, additional pharmacovigilance studies are necessary to better understand the relationship between opioid sedation protocols, polypharmacy, and ADE occurrence in critically ill patients.

## Data Availability

The original contributions presented in the study are included in the article/Supplementary Material, further inquiries can be directed to the corresponding author.
